# Supplementary material for: An integrated clinical and genetic model for predicting risk of severe COVID-19: A population-based case–control study
Source: PLoS One. 2021 Feb 16;16(2):e0247205. doi: 10.1371/journal.pone.0247205 (PMC7886160; doi:10.1371/journal.pone.0247205)
Supplement: S1 Table — (PDF) [file pone.0247205.s001.pdf]

S1 Table. Single-nucleotide polymorphisms.

Chromosome	ID	Reference allele	Risk allele	Risk allele odds ratio	Risk allele frequency
1	rs12745140	G	A	2.21	0.11
1	rs12083278	G	C	1.83	0.70
1	rs2765013	T	C	2.33	0.92
1	rs2274122	G	A	1.78	0.80
1	rs10873821	T	C	1.75	0.77
2	rs6714112	A	C	2.19	0.86
2	rs2270360	C	A	1.72	0.71
3	rs1504061	C	G	2.52	0.06
3	rs17317135	A	G	2.65	0.94
3	rs1868132	C	T	1.97	0.10
3	rs6440031	A	G	2.19	0.89
4	rs3774881	C	T	1.87	0.85
4	rs3774882	G	C	2.38	0.92
4	rs6810404	A	C	1.58	0.51
4	rs35540967	T	C	2.46	0.07
4	rs115162070	A	G	2.69	0.92
4	rs11729561	C	T	2.25	0.92
4	rs112641600	T	C	2.17	0.90
5	rs62377777	C	T	1.75	0.79
5	rs4240376	T	G	1.75	0.80
5	rs10039856	T	C	2.16	0.91
5	rs2220543	A	T	1.73	0.71
5	rs113791144	C	T	2.27	0.06
6	rs6933436	A	C	1.62	0.28
6	rs10755709	G	A	1.66	0.69
6	rs140247774	C	T	2.60	0.06
6	rs16873740	T	A	2.04	0.12
6	rs9386484	A	T	1.86	0.75
8	rs118072448	C	T	2.38	0.91
8	rs10808999	A	G	1.89	0.86
8	rs13282163	C	A	2.39	0.93
8	rs11779911	A	C	1.63	0.66
8	rs2010843	T	C	1.59	0.55
9	rs3895472	T	C	2.42	0.91
9	rs12236000	C	G	2.44	0.93
9	rs7027911	G	A	1.59	0.44
10	rs71481792	T	A	1.71	0.38
10	rs2091431	A	G	1.62	0.71
10	rs1892429	G	A	1.90	0.79
10	rs10793436	T	G	1.68	0.68
10	rs1441121	A	T	1.59	0.56
11	rs10766439	G	A	1.57	0.39
12	rs11613792	G	A	2.13	0.84
12	rs12823094	T	A	1.70	0.26
13	rs1984162	A	G	1.65	0.26
13	rs12871414	T	C	1.64	0.72

Chromosome	ID	Reference allele	Risk allele	Risk allele odds ratio	Risk allele frequency
14	rs2238187	A	G	1.70	0.36
14	rs12587980	C	T	1.54	0.39
15	rs12593288	T	C	1.79	0.78
15	rs2229117	C	G	1.93	0.87
16	rs72803978	G	A	2.68	0.94
17	rs34761447	T	C	2.17	0.89
17	rs178840	A	G	1.70	0.75
18	rs12958013	C	T	1.94	0.85
19	rs8105499	A	C	1.62	0.69
19	rs60744406	A	G	1.64	0.61
19	rs10411226	A	G	1.77	0.24
21	rs2252109	T	A	1.60	0.49
22	rs5757427	A	T	1.80	0.63
22	rs7290963	G	T	1.59	0.45
22	rs11090305	T	C	1.84	0.18
22	rs62220604	A	G	1.69	0.71
3	rs11385942	G	GA	1.77	0.09
9	rs657152	C	A	1.32	0.35

Note: All SNPs are from release 2 of the COVID-19 Host Genetics Initiative meta-analysis of hospitalisation vs non-hospitalisation [1, 2], except for rs11385942 and rs657152, which are from Ellinghaus et al [3].

1. COVID-19 Host Genetics Initiative. COVID-19 Host Genetics Initiative: results [Internet]. 2020 [cited 2020 May 13]. Available from: https://www.covid19hg.org/results/.
2. COVID-19 Host Genetics Initiative. The COVID-19 Host Genetics Initiative, a global initiative to elucidate the role of host genetic factors in susceptibility and severity of the SARS-CoV-2 virus pandemic. Eur J Hum Genet. 2020;28(6):715-8. Epub 2020/05/15. doi: 10.1038/s41431-020-0636-6.
3. Ellinghaus D, Degenhardt F, Bujanda L, Buti M, Albillos A, Invernizzi P, et al. Genomewide association study of severe Covid-19 with respiratory failure. N Engl J Med. 2020;383(16):1522-34. Epub 2020/06/20. doi: 10.1056/NEJMoa2020283.
