# Supplementary material for: An integrated clinical and genetic model for predicting risk of severe COVID-19: A population-based case–control study
Source: PLoS One. 2021 Feb 16;16(2):e0247205. doi: 10.1371/journal.pone.0247205 (PMC7886160; doi:10.1371/journal.pone.0247205)
Supplement: S2 Table — (PDF) [file pone.0247205.s002.pdf]

S2 Table. Disease definitions.

Disease	ICD9	ICD10
Asthma	493*	J45*, J46
Autoimmune (rheumatoid arthritis/ lupus/psoriasis)	6954, 696*, 7100, 714, 7140*, 7142*	J990, L40*, L41*, M05*–M07*, M32*
Cancer - haematological	200*–208*	C81*-C86*, C88*, C90*-C96*
Cancer - non-haematological	140*–165*, 169*–175*, 179*–195*, 196*–199*	C00*–C26*, C30*–C34*, C37*–C58*, C60*–C80*, C97*
Cerebrovascular disease	430*-438*	G46*, I60*-I69*
Diabetes	250*	E10*–E14*
Heart disease	413*–416*, V422, V432–V434	I20*–I25*, I48*, Z95*
Hypertension	401*, 405*, 6420–6422	I10*, I15*, O10*
Immunocompromised	V420, V421, V426, V427, V429, 042, 043, 044, 279, 2790*	B20*–B24, D80*–D84*, Z940–Z944, Z949
Kidney disease	585*	N18*
Liver disease	571*	K70*–K77*
Respiratory disease (excluding asthma)	494*–496*, 500*, 501*-508*, 491*, 492*, 496*	J60*-J70*, J80*–J82, J84*–J86*, J90–J96*, J98*, J41*–J44*
