# Supplementary material for: An integrated clinical and genetic model for predicting risk of severe COVID-19: A population-based case–control study
Source: PLoS One. 2021 Feb 16;16(2):e0247205. doi: 10.1371/journal.pone.0247205 (PMC7886160; doi:10.1371/journal.pone.0247205)
Supplement: S3 Table — (PDF) [file pone.0247205.s003.pdf]

S3 Table. Sensitivity analysis.

Sensitivity analyses including the 20 cases and 41 controls with no hospital data showed no change to the associations for SNP score, age ABO blood type, autoimmune disease, haematological cancer and diabetes. There was a small increase in effect size for ethnicity, hypertension and non-haematological cancer. There was a small decrease in effect size for gender. The conclusions of the analysis are unchanged in the sensitivity analysis.

Variable	Categories	Adjusted odds ratio	95% confidence interval	P value
SNP score	% risk alleles	1.18	1.15 to 1.22	<0.001
Age group (years)	50–59	–		
	60–69	0.92	0.67 to 1.26	0.60
	70+	1.71	1.26 to 2.32	0.001
Gender	Female	–		
	Male	1.10	0.87 to 1.39	0.41
Ethnicity	White	–		
	Other/Missing	1.48	1.04 to 2.10	0.03
ABO blood type	O	–		
	A	0.82	0.64 to 1.05	0.12
	B	1.16	0.78 to 1.72	0.46
	AB	0.42	0.22 to 0.79	0.007
Autoimmune (rheumatoid arthritis/ lupus/psoriasis)	No	–		
	Yes	2.20	1.20 to 4.02	0.01
Cancer – haematological	No	–		
	Yes	2.84	1.11 to 7.24	0.03
Cancer – non-haematological	No	–		
	Yes	1.48	1.07 to 2.05	0.02
Diabetes	No	–		
	Yes	1.64	1.16 to 2.31	0.005
Hypertension	No	–		
	Yes	1.41	1.08 to 1.84	0.01
Respiratory disease (excluding asthma)	No	–		
	Yes	3.50	2.59 to 4.72	<0.001
