# Supplementary material for: An integrated clinical and genetic model for predicting risk of severe COVID-19: A population-based case–control study
Source: PLoS One. 2021 Feb 16;16(2):e0247205. doi: 10.1371/journal.pone.0247205 (PMC7886160; doi:10.1371/journal.pone.0247205)
Supplement: S4 Table — (PDF) [file pone.0247205.s004.pdf]

S4 Table. Model with age group and gender.

Variable	Categories	Adjusted odds ratio	95% confidence interval	P value
Age group (years)	50–59	–		
	60–69	1.28	0.96 to 1.69	0.09
	70+	2.78	2.16 to 3.57	<0.001
Gender	Female	–		
	Male	1.29	1.05 to 1.60	0.02
