# Supplementary material for: An integrated clinical and genetic model for predicting risk of severe COVID-19: A population-based case–control study
Source: PLoS One. 2021 Feb 16;16(2):e0247205. doi: 10.1371/journal.pone.0247205 (PMC7886160; doi:10.1371/journal.pone.0247205)
Supplement: S5 Table — (PDF) [file pone.0247205.s005.pdf]

S5 Table. Model with clinical risk factors.

Variable	Categories	Adjusted odds ratio	95% confidence interval	P value
Age group (years)	50–59	–		
	60–69	0.91	0.67 to 1.23	0.53
	70+	1.56	1.16 to 2.08	0.003
Gender	Female	–		
	Male	1.09	0.87 to 1.37	0.46
Ethnicity	White	–		
	Other/Missing	1.04	0.74 to 1.47	0.82
ABO blood type	O	–		
	A	0.94	0.74 to 1.20	0.62
	B	1.23	0.83 to 1.80	0.30
	AB	0.57	0.31 to 1.07	0.08
Autoimmune (rheumatoid arthritis/ lupus/psoriasis)	No	–		
	Yes	1.72	0.97 to 3.05	0.06
Cancer – haematological	No	–		
	Yes	2.75	1.13 to 6.68	0.03
Cancer – non-haematological	No	–		
	Yes	1.27	0.94 to 1.73	0.12
Diabetes	No	–		
	Yes	1.47	1.06 to 2.03	0.02
Hypertension	No	–		
	Yes	1.36	1.05 to 1.75	0.02
Respiratory disease (excluding asthma)	No	–		
	Yes	3.54	2.66 to 4.72	<0.001
